# Preparation and characterization of nanosized P(NIPAM-MBA) hydrogel particles and adsorption of bovine serum albumin on their surface

**DOI:** 10.1186/1556-276X-7-519

**Published:** 2012-09-24

**Authors:** Xiaoli Zhu, Xiangling Gu, Lina Zhang, Xiang-Zheng Kong

**Affiliations:** 1 College of Chemistry and Chemical Engineering, University of Jinan, Jinan, 250022, China; 2 Department of Medicine, Dezhou University, Dezhou, Shandong, 253023, China

**Keywords:** *N*-isopropylacrylamide, Precipitation polymerization, Hydrogel nanoparticles, Bovine serum albumin

## Abstract

Thermosensitive polymer hydrogel particles with size varying from 480 to 620 nm were prepared through precipitation copolymerization of *N*-isopropylacrylamide with *N,N*′-methylenebisacrylamide (MBA) in water with ammonium persulfate as the initiator. Only polymer hydrogels without any coagula were obtained when MBA concentration in the monomer mixture was kept between 2.5 and 10.0 wt%; with increased MBA concentration, the monomer conversion was enhanced, the size of the hydrogels was increased, and their shrinking was lessened when heated from 25°C to 40°C. Bovine serum albumin adsorption on the surface of the hydrogels of different MBA content was measured at different pH levels and under different temperatures. The results demonstrated that the adsorption of the protein on the hydrogels could be controlled by adjusting the pH, the temperature of adsorption, and the crosslinking in the hydrogels. The results were interpreted, and the mechanisms of the polymerization were proposed.

## Background

Poly(*N*-isopropylacrylamide), PNIPAM, has been known as a thermosensitive material. It exhibits a volume phase transition temperature (VPTT) around 32°C. Heskins et al. [1] were probably the first to report a study on its solution behaviors and ascribed the thermodynamic properties to an entropy effect. Since then, numerous studies on PNIPAM and its related polymers have been reported [2-14], and these materials have been widely used in different areas of high technology, particularly in biomedical areas. Since the report by Pelton et al. [3], the latex particles of PNIPAM and its copolymers have been used as supports for various biomolecules including proteins [4], enzymes [5], granulocyte [6], oligodeoxyribo-nucleotide [7] and antibodies [8]. They are also used to the controlled drug delivery systems [9]. Magnetic latex particles of these materials and their application in biomedical field have been also reported [10].

Nano- or microgels of PNIPAM and of its copolymers can be prepared by heterophase polymerizations with different crosslinkers in aqueous phase. The possible processes include precipitation polymerization, water-in-oil emulsion polymerization [11], and micro-emulsion polymerization [12]. Among these, precipitation polymerization is probably the most frequently used technique [14-17]. Nagaoka et al. synthesized PNIPAM hydrogels by radiation polymerization [18]. Kawaguchi et al. [4] prepared monodisperse PNIPAM hydrogel nanoparticles and used them as adsorbent for human gamma globulin. Through copolymerization of NIPAM with ionic monomers, pH and temperature sensitive hydrogels were also obtained, and this way the VPTT [19,20] of the resultant materials was often shifted to a higher temperature. Kratz et al. prepared anionic hydrogels through copolymerization of NIPAM with acrylic acid [21]. Elaissari et al. prepared cationic hydrogels by copolymerization of NIPAM with aminoethyl methacrylate hydrochloride in presence of a cationic initiator [22].

In a previous work, we have prepared monodisperse particles of poly(trihydroxy-methyl propane triacrylate-styrene), i.e., P(TMPTA-St), in ethanol and ethanol/water mixture [23,24]. In this article, precipitation polymerization of NIPAM in water with *N,N*′-methylenebisacrylamide as the crosslinker and ammonium persulfate as the initiator was conducted. Thermosensitive hydrogel particles with sizes of 480 nm or larger were prepared, and adsorption of bovine serum albumin (BSA) on their surface was also studied.

## Methods

### Materials

Before use, *N*-isopropylacrylamide (NIPAM, CP grade, from TCI Development Co., Ltd., Shanghai, China) was recrystallized in hexane (AR, Tianjin Fuyu Fine Chemicals Co., Ltd., Tianjin, China), *N,N*′-methylenebisacrylamide (MBA, AR, Tianjin Kemiou Chemical Reagent Co., Ltd., China) recrystallized in methanol (AR, Tianjin Fuyu Chemicals), and ammonium persulfate (APS, AR, Tianjin Fuyu Chemicals) in water, respectively. Bovine serum albumin (AR, Shanghai Lanji Science and Technology Development Co., Ltd., China; M_*w*_ = 67 kDa; isoelectric point, IEP 4.7) was stored in a refrigerator. Coomassie Brilliant Blue (G-250, AR, Shanghai Yixin Chemical Co., Ltd., Beijing, China) was used as received.

### Preparation of hydrogel particles based on NIPAM

Hydrogel particles of P(NIPAM-MBA) were prepared by free radical precipitation polymerization. In a typical run, 1.8 g of NIPAM, 0.2 g of MBA, the crosslinker monomer, and 90.0 mL of deionized water were charged into a glass bottle of 120-mL capacity, which was located into a thermostat shaker at room temperature and shaken until the complete dissolution of the monomers, followed by N_2_ purge of the reaction system for 5 min and addition of 0.04 g of APS dissolved in 8 mL of water. The temperature of the thermostat shaker was then rapidly risen to 70°C within 1 h while keeping the shaking at 120 osc·min^−1^. The reaction was allowed to run for 6 h. The resultant polymers were separated out by repeated centrifugation at room temperature and washing with deionized water three times. Hydrogel particles with different monomer composition were prepared by varying the crosslinker amount.

### Adsorption and desorption of BSA

To test the adsorption of BSA on the hydrogel particles at different pH levels, 1.5 g of dried particles was added into 150 mL of BSA aqueous solution of 0.5 g·L^−1^ concentration. The mixture was shaken up and evenly divided into three portions. Every portion was put in a bottle of 50 mL capacity with their pH adjusted respectively to 4, 7, and 11. The bottles were located into a thermostat shaker with temperature set at 40°C. BSA adsorption was processed for 2 h at a shaking speed of 120 osc·min^−1^. Of the mixture, 20 mL was taken out from the bottle and centrifuged for 5 min at 12,000 rpm to separate the particles out from the mixture. Of the supernatant, 400 μL was added into a 4-mL aqueous solution of Coomassie Brilliant Blue G-250 of known concentration. The amount of BSA remaining in the supernatant was determined by ultraviolet spectrophotometer (UV-2450, Shimadzu Corporation, Japan), and the amount of BSA adsorbed was thus obtained. BSA adsorption at 20°C was conducted by lowering the temperature of the mixture at the end of the adsorption at 40°C to 20°C, a temperature below the VPTT of the hydrogels, where they became hydrophilic and were highly hydrated. The bottles were kept shaken for another 2 h. The amount of BSA desorbed was determined the same way as described above.

### Characterization

At end of the polymerization, polymers were present under two forms: polymer particles and that remaining soluble in water. The yield of the polymer particles was determined gravimetrically by weighing the dried polymer powder collected upon centrifugation, and that of the soluble polymers was obtained by drying the supernatant. The sum of the two yields was considered as the total monomer conversion. Morphology of the polymer particles was examined using scanning electron microscope (SEM, QUANTA FEG-250, FEI Company, USA), and their number average size (*D*_n_) was calculated by counting about 200 particles on the SEM pictures. Their size and size distribution (PDI) were also measured using a dynamic light scattering instrument (Nano-ZS, Malvern Instruments Ltd., UK). Variation of light transmittance of the samples at different temperature was followed using a photometer (Mod-662, Metrohm Ltd., Switzerland) at 565-nm wavelength.

## Results and discussion

### Effect of MBA content on particle formation and morphology

In precipitation polymerization, a commonly known fact is that the crosslinker monomer plays an important role in particle formation [24]. In order to establish an appropriate protocol for this precipitation polymerization, a set of experiments was first carried out using different MBA amounts, while the concentration of the monomers was kept constant at 2.0 wt%. Yields of the polymer particles and soluble polymer and monomer conversion were determined. The hydrodynamic size of the hydrogel particles was measured at 25°C (*D*_1_) and 40°C (*D*_2_), the ratio D_1_/D_2_, denoted as their shrinking ratio, was obtained. The results are given in Table 1. 

**Table 1:** Precipitation polymerization of NIPAM-MBA at 70°C with different MBA amount

**MBA (wt%)**	***D***_**1**_^**a**^**(nm)**	***D***_**2**_^**a**^**(nm)**	***D***_**1**_**/*D***_**2**_	**Particle yield (%)**	**Soluble polymer (%)**	**Monomer conversion (%)**	***D***_**n**_**(nm)**	***N***_**p**_	**Observations**
								**(10**^**14**^**L**^**−1**^**)**	
2.5	1,110	498	2.23	87.14	12.35	99.49	487	3.079	Stable latex
5.0	1,190	599	1.99	90.74	8.67	99.41	564	2.064	White precipitate
7.5	1,004	613	1.64	91.48	7.82	99.30	602	1.711	As above
10.0	1,040	716	1.45	91.78	7.06	98.84	620	1.572	As above
12.5	-	-	-	-	-	-	-	-	Coagula

^a^*D*_1_ and *D*_2_ are the sizes measured by dynamic light scattering at 25°C and 40°C, respectively. *N*_p_, number of particles.

It was found that stable latex was obtained when 2.5 wt% of MBA was used, and the resultant latex remained stable even after centrifugation at 12,000 rpm for 1 h. With MBA content increased to 5.0 wt%, the polymer precipitated out from the reaction mixture; phase separation occurred immediately upon the bottle shaking was stopped. Coagulation appeared when the MBA amount further increased to 12.5 wt%. It is commonly agreed that particle nucleation in this precipitation polymerization is done by oligomers grown up to a critical length, where they become insoluble and precipitate out to form the primary particles. This nucleation proceeds quite fast, and abundant primary particles can be formed in a short period. Once this period of nucleation is completed, particle growth is continued by an entropy mechanism as proposed by Stöver et al. [25], by which the monomers and the oligomers, prior to reaching their critical length, polymerize with the residual double bonds on the surface of the particles, instead of by adsorption of oligomers after reaching their critical length (i.e., by enthalpy mechanism).

As described in the ‘Methods’ section, the size of the particles was determined through dynamic light scattering at 25°C (Table 1, *D*_1_) and 40°C (*D*_2_), two different temperatures across the VPTT (about 32°C) of the hydrogels. It is obvious that the particles at 25°C were fully swelled, owing to strong interaction of their polymer chains with water; and at 40°C, the particles were deswelled to squeeze water molecules out because they were at the temperature above their VPTT. The particle size determined at 25°C (*D*_1_) was, therefore, significantly larger than that at 40°C (*D*_2_). Table 1 listed also the particle size obtained from SEM (*D*_n_). The result demonstrated that *D*_n_ was slightly smaller than the hydrodynamic size of the particles at deswelled state (*D*_2_) regardless of MBA amount used. This has been often observed and attributed to the fact that these particles possess hydrophilic polymer segments or end chain groups on their surface. It is commonly known that light scattering in determination of latex particles does not make distinction between the veritable polymer particles and the hydrated layer on their surface; the size of the particles, thus, determined is therefore larger than their size at dry state obtained from TEM or SEM [26,27]. Obviously, the size value determined at 40°C (*D*_2_) in this work reflected the real size of the particles, i.e., the hydrogel particle size from SEM (*D*_n_). The size of the particles was of nano- or submicron scale.

Results in Table 1 demonstrated that, independent of the methods used for their determination, the size of the nanospheres was increasing with MBA content in the monomers, in agreement with the reported observation [28]. This increase in particle size can be also interpreted based on the mechanisms of particle nucleation and growth. It is believed that the use of a crosslinker monomer, MBA in the present case, promoted significantly the increase in molecular weight of the oligomers, and therefore accelerated the formation of the primary particles. However, coalescence or combination of the primary particles might well occur when a too large number of primary particles was formed in a short period of time [23]. This coalescence would result in particles with larger size, reducing the number of particles in the polymerization system, accompanied by formation of no spherical particles, particularly if a high concentration of crosslinker monomer was used and this coalescence occurred at high monomer conversion, where the subsequent polymerization of the remaining monomers was not enough to smooth the coalesced primary particles. Based on this consideration, the increase in particle size with increase in MBA is well understood so is the decrease in the number of particles (*N*_p_).

Data in Table 1 reveal that lower particle yields were observed with lower MBA content. This yield was slightly enhanced with increased MBA up to 7.5 wt% and remained practically unchanged afterwards. It is known that the use of crosslinker is a must in the precipitation polymerization, aiming at fabrication of uniform particles [23,24,29]. Crosslinker monomers greatly boost the molecular weight of the oligomers and promote the formation of the primary particles at beginning of the polymerization. Without the use of a crosslinker monomer, oligomers remain soluble in the polymerization medium for a longer time, and their accumulation leads often to coagula formation with progress of polymerization. With 2.5 wt% of MBA, the lowest in all the runs given in Table 1, a lowest particle yield of 87.14% was obtained along with a highest yield of soluble polymers of 12.35%. This was in good agreement with the above mechanisms of particle nucleation and growth. The decrease in particle yields was compensated by an increase in soluble polymers, and by consequence, high monomer conversion was achieved for all runs regardless of the MBA amount used.

**Figure 1:**
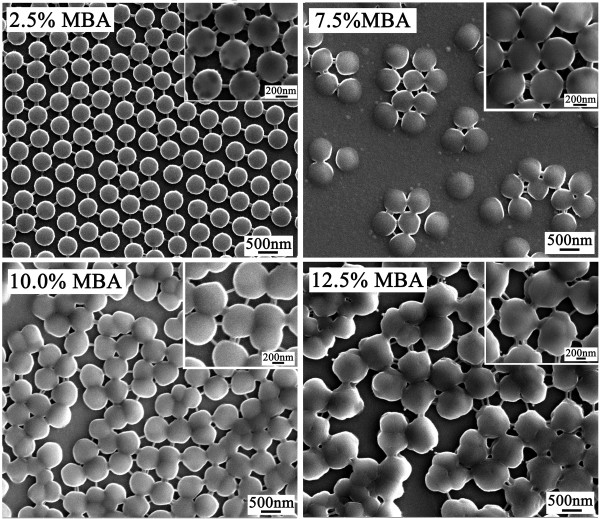
** SEM images.** SEM micrographs of P(NIPAM-MBA) particles with different MBA amounts.

For a visual illustration, the final products of the polymerizations were examined under SEM. Selected micrographs are displayed in Figure 1 which shows that polymer particles of high uniformity were formed with 2.5 wt% of MBA. With increase in MBA concentration, the uniformity of the particles was obviously deteriorated, and aggregation of the particles was well discernible in samples with 10.0 wt% of MBA. With 12.5 wt% of MBA, practically, only large particles consisting of small ones with irregular forms were present, with obvious presence of coalesced small particles. Note that the morphology evolution of the particles with increased MBA in Figure 1 provided a solid support to the interpretation on the variations of the size and the number of the particles given in Table 1.

It is also worth to note that abundant interlinkage was present between the nanospheres (Figure 1, inserted photos in particular), a distinct difference from the polymer particles prepared through emulsion polymerization. This observation was believed to be caused by the relatively heavy crosslinking in the particle polymer, and therefore attributable to the use of MBA in the polymerization. It is obvious that PNIPAM alone cannot be crosslinked. Polymer chains extending to water phase without crosslinking, if any, must be closely packed on the surface when drying up. It was reported that MBA polymer was more hydrophilic than NIPAM [14,30], which may result in the particles with higher crosslinking at their surface layer rather than in their core. This MBA enrichment on the surface of the particles contributed to the formation of the interlinkage between the particles.

### Thermo-sensitive properties of P(NIPAM-MBA) particles

**Figure 2:**
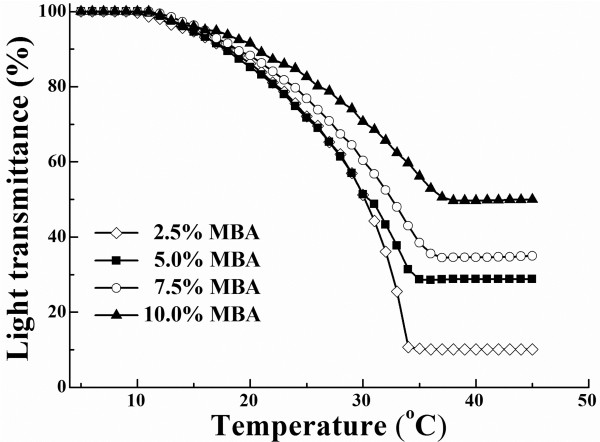
** Light transmittance of P(NIPAM-MBA) dispersions.** Dependence of light transmittance of P(NIPAM-MBA) dispersions on temperature.

One of the important features of the P(NIPAM-MBA) particles is their thermosensitive property, characterized by a VPTT at about 32°C. To understand the impact of MBA on the thermosensitive properties, the samples were first diluted to low solids (0.1 wt%) and subjected to light transmittance test by increasing the temperature from 5°C to 45°C by increment of 1°C. The results are depicted in Figure 2.

It is seen that, below 10°C, the light transmittance in all the samples was 100%, regardless of the MBA content in the copolymers, indicating that all samples were transparent liquid and that the particles were fully swelled by water. However, when the temperature was risen to 15°C and above, the transmittance of the samples started to decline at rates dependent on the particle composition. PNIPAM homopolymer is known to have a VPTT at 32°C [2,14]. Figure 2 demonstrated that incorporation of MBA rendered a broadened VPTT and raised its average value in the cross-linked copolymers.

For thermosensitive hydrogels, increasing the temperature from a point below its VPTT, where the particles are fully swelled with water, is a process in which the water molecules contained in the particles are expelled out due to the disruption of hydrogen bonding between water molecules and the hydrophilic amide groups, causing at the same time the packing up of the hydrophobic isopropyl groups in the polymer chains. This process makes the particles shrink to a smaller size. In Table 1, the diameters of the particles at 25°C (*D*_1_) and 40°C (*D*_2_) were determined for each samples of different MBA, which indeed confirmed the particle volume shrinking. The shrinking ratio, *D*_1_/*D*_2_, was rapidly decreasing with the increase in MBA, obviously owing to the increased crosslinking in the particles.

The influence of MBA amount was also clearly seen in Figure 2. While the sample with 2.5 wt% of MBA, the lowest among the four samples, was characterized by a most rapid decline in light transmittance reached a lowest value in a shortest time, the one with 10.0 wt% of MBA, the highest MBA amount, was characterized by a slowest decline with a highest final value of light transmittance reached in a longest period of time. The corresponding curves for the other samples lay in between these two samples, in the order of their MBA content. As aforementioned, the observed decline of the light transmittance was a reflection of the dehydration in the water-swollen hydrogel particles, owing to disruption of hydrogen bonding between water molecules and amide groups on polymer chains. This dehydration proceeded the way likely to squeeze water out from the fully hydrated particles by packing up more tightly the polymer chains. It is easy to conceive that the water squeezing was easier and more complete in a particle with lessened crosslinking, and it became harder when the particles were crosslinked at a higher degree; and there was also less water available at start of the experiment (at 5°C) due to the high crosslinking. This was exactly the case shown in Figure 2.

### Evolutions of particle and soluble polymer yields during polymerization

**Figure 3:**
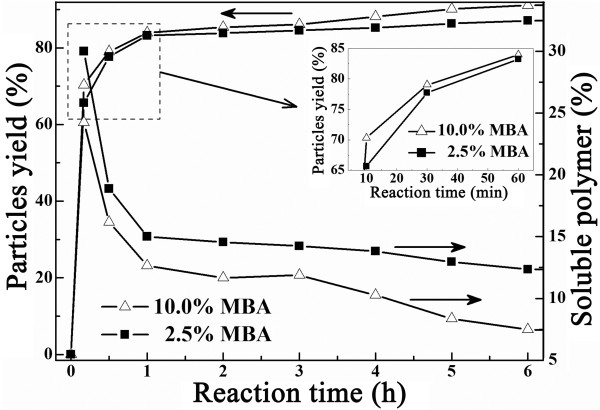
** Particle yields and soluble polymers.** Evolutions of particle yields and soluble polymers in NIPAM-MBA polymerization.

The evolutions of the yields of the particles and of the soluble oligomers in the course of the polymerization were also followed in order to understand the process of the polymerization. These yields in two runs, one with high MBA of 10.0 wt% and another with low MBA of 2.5 wt%, are displayed in Figure 3. Note that the sum of the two yields gave the monomer conversion. Figure 3 revealed that the polymerization proceeded quite fast; the conversion reached about 95% within 10 min. At this time, a large portion of the conversions was contributed by the soluble oligomers: about 30% for the run with 2.5 wt% of MBA, and this value slightly lowered to about 25% for the run with 10.0 wt% of MBA. Within 1 h of polymerization time, all of the monomers were practically polymerized, i.e., the highest conversion (100%) was attained. At the same time, the proportion of the soluble polymers relative to all converted monomers dramatically dropped to about 14% and 12% for the runs with 2.5 wt% and 10.0 wt% of MBA, respectively. As to the particle yield, little change was detected with further progress in polymerization for the run with lower MBA, and a slight increase was observed for the run with higher MBA amount of 10.0 wt%, concomitantly with a slight decrease in the yield of the soluble polymers. After all, it seemed the amount of MBA has little effect on the rate of polymerization, although the run with 10.0 wt% of MBA was seen to have a slightly higher yield for the particles (see the inserted figure in Figure 3).

### Influence of initiator concentration

It is known that the concentration of initiator has important effects on the process of polymerization, particularly on the particle formation [23,24]. A set of polymerizations was carried out at 70°C with different concentrations of the initiator APS. Monomer conversion, particle size, and the yields of the soluble polymers were all determined (Table 2). 

**Table 2:** Precipitation polymerization of NIPAM-MBA with varied APS levels

**APS (wt%)**	***D***_**1**_^**a**^**(nm)**	***D***_**2**_^**a**^**(nm)**	**PDI (40°C)**	**Particle yield (%)**	**Soluble polymer (%)**	**Monomer conversion (%)**
0.5	860	632	0.112	79.17	4.79	83.96
1.0	873	665	0.140	84.52	5.70	90.22
1.5	989	699	0.201	87.93	7.07	95.00
2.0	1,040	716	0.198	91.78	7.06	98.84
2.5^b^	5,156	1,583	0.379	91.18	7.55	98.73
3.0^b^	3,965	1,723	0.399	91.24	7.59	98.83

^a^*D*_1_ and *D*_2_ are the sizes of the particles determined by light scattering at 25°C and 40°C, respectively. ^b^In these two runs, particles were observed with part of the polymer coagula. Only particles were obtained in the rest of the runs, without coagula.

**Figure 4:**
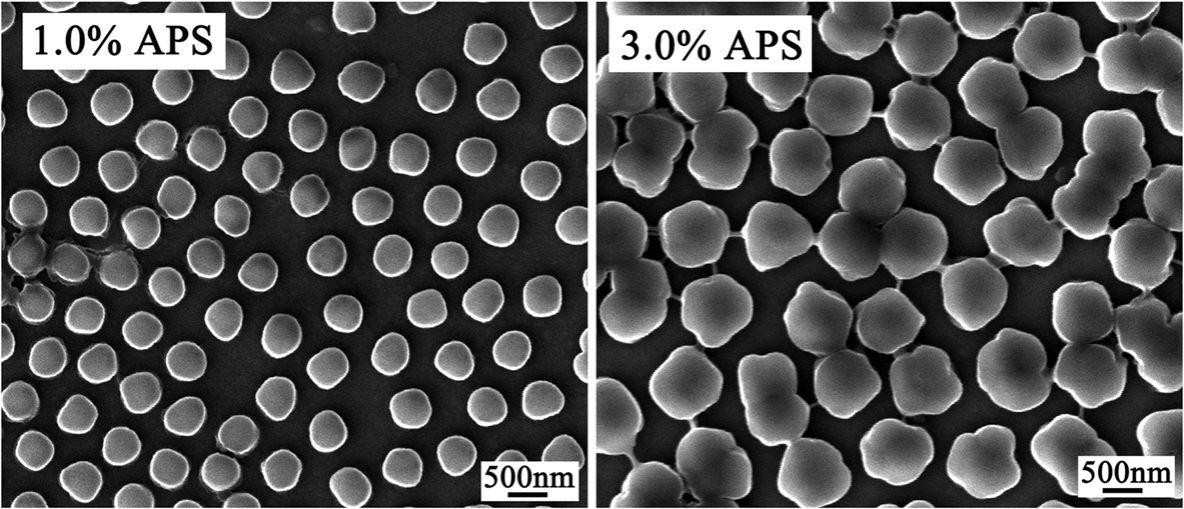
** SEM images.** SEM micrographs of P(NIPAM-MBA) particles prepared with varied APS concentrations.

An obvious remark was that the monomer conversion was low at low APS concentration and gradually increasing in accordance with the increase in APS concentration. The proportion of the soluble polymers was also increasing as one could have expected. At low APS content, the initiator was insufficient to quickly start the initiation, the number of free radicals was at a low level, and so was that of oligomers by consequence, as seen through the yield of the soluble polymers. With more APS, more oligomers must be produced, leading to a higher concentration of the soluble polymers. The yield of the nano-particles, however, showed a different behavior. Below 2.0 wt% of APS, this yield was increasing with APS increase, and a slight decrease was detected after reaching a maximum at 2.0 wt% of APS. This indicates that APS was better to be limited to 2.0 wt% in order to maintain a high yield for the hydrogel particles with a high monomer conversion while keeping the soluble polymers at a low level. Selected SEM pictures are shown in Figure 4, which demonstrate that with high APS concentration, the particles became no spherical, owing to aggregation or coalescence of the primary particles when a large number of them were formed as discussed above. In the present case, this increase in number of the primary particles was caused by the use of a high concentration of APS, rather than by an accelerated nucleation of the particles due to high concentration of MBA as seen in Table 1.

### Adsorption and desorption of BSA on poly(NIPAM-MBA) hydrogel particles

**Figure 5:**
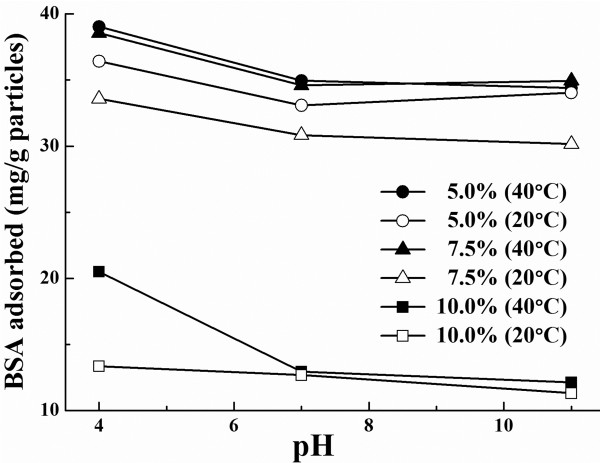
** BSA absorption on P(NIPAM-MBA) particles.** Amount of BSA absorbed on P(NIPAM-MBA) particles of different MBA content at varied pH and temperature (the percentages in the figure referred to MBA content).

Among all the smart polymeric materials, that based on NIPAM is the most widely studied. These materials can respond with shape and volume changes to small external stimuli, including temperature, pH, ionic strength, and magnetic field [14,31], and have been used in biomedical and biological applications such as enzyme immobilization, cell sorting, protein adsorption and purification, and drug delivery [32]. The adsorptions of BSA on P(NIPAM-MBA) particles prepared with different MBA content at 40°C and at 20°C in different pH were determined; the results are shown in Figure 5. It is clearly seen that the amount of BSA adsorbed on the particles at 40°C was always higher than that at 20°C, regardless of the medium pH in the test and the MBA amount used in the preparation of the polymers. This increase in the amount of adsorbed proteins has been often reported [4,29,30,33,34] and was often attributed to the enhanced hydrophobicity of the particle surface when water was expelled out at temperature above the VPTT. Figure 5 revealed also that more BSA was adsorbed at lower pH, regardless of their crosslinking, which implies that this adsorption was governed not only by hydrophobic interaction but also by electrostatic interactions. By lowering the pH from 7 to 4, the increase in BSA adsorption was remarkable, whereas the decrease was very slight when pH was increased from 7 to 10. Knowing that BSA has an IEP of 4.7 [35], BSA molecules bore, therefore, negative charges at pH above 7 and were positively charged below its IEP. Whereas for the particles, they were assumingly negatively charged because of the use of the anionic initiator APS, with -SO_4_^−^ groups at the terminals of the polymer chains. When the pH of the medium was at 4, below its IEP, there existed, besides the hydrophobic adsorption, an electrostatic interaction between the positive charged BSA molecules and the negatively charged particle surface [31]. When adsorption on particles of different MBA content was examined, it is seen that BSA adsorption decreased with MBA content increase, i.e., with increased crosslinking in the particles regardless of the pH. While the decrease was not important when MBA increased from 5.0 to 7.5 wt%, this decrease was significant with MBA increase from 7.5 to 10.0 wt%. It is reported that MBA polymer was assumingly more hydrophilic than NIPAM at the temperature of the polymerization [30], which might result in the particles with higher crosslinking at their surface layer than in the core, which seemed confirmed by the presence of interlinks between the particles as seen from Figure 1. This implies that the particle surface was likely more hydrophilic due to the presence of higher MBA containing segments, leading to therefore lower BSA adsorption when more MBA was used. Another possibility associated to the crosslinking effect is that, at lower crosslinking, the hydrogels were more swelled or less packed; BSA molecules were also possible to diffuse inside of the hydrogels and to make therefore the hydrophobic sites inside the hydrogels accessible for BSA adsorption. 

All these results demonstrate that the adsorption of BSA molecules could be controlled by adjusting the crosslinking of the polymers in the particles, the pH, and the temperature of the experiments.

## Conclusions

Nano and micro-sized hydrogel particles based on P(NIPAM-MBA) were prepared by precipitation polymerization in water at 70°C. Results revealed that the monomers polymerized very fast, the conversion reached about 95% within 10 min, and the proportion of the soluble polymers dramatically dropped down to about 12% at 1 h of polymerization time. The yields of the particles and their size were increased with MBA increase up to 7.5 wt%. Incorporation of MBA in the thermosensitive material caused a broadened VPTT and raised its average value in the cross-linked copolymers. Size determinations of the particles at temperatures above and below their VPTT confirmed a volume shrinking for the particles above their VPTT and the shrinking was rapidly weakened with increase in MBA, owing to the obviously increased crosslinking in the particles. As to the influence of the initiator APS on the polymerization, an obvious remark was that monomer conversion and the soluble polymers were both gradually increasing in accordance with increase in APS. Tests on BSA adsorption revealed that the amount of BSA adsorbed on the particles above its VPTT was always higher than that below. BSA adsorption was also enhanced at pH below the IEP of BSA. However, BSA adsorption decreased with increased crosslinking in the particles regardless of the pH. While the decrease was not important when MBA increased from 5.0 to 7.5 wt%, it was significant with MBA increased from 7.5 to 10.0 wt%.

## Competing interests

The authors declare that they have no competing interests.

## Authors’ contributions

XG and LZ performed the experiment. XZ analyzed the data and drafted the manuscript. XZK designed the study and finalized the manuscript. All authors read and approved the final manuscript.

## Authors’ information

XZ received her BSc degree in Chemistry in 1996 and MSc degree in Polymer Chemistry and Physics from Shandong University in 1999. After being employed as a lecturer in Shandong Institute of Light Industries for 3 years, she continued her study in Shandong University and obtained her PhD degree in Materials Science in 2005. She is currently an associate professor at the University of Jinan. Her main research interests focus mainly on preparations of functional polymer microspheres with different shapes through emulsion polymerization and precipitation polymerization, and aqueous dispersions of polyurethanes modified with polysiloxanes and/or polyacrylates. She has published 60 papers and holds four Chinese invention patents.
